# Morphological and molecular identification of *Hyalomma* ticks in Kazakhstan reveals new regional records of *H. dromedarii, H. rufipes*, and *H. asiaticum*

**DOI:** 10.3389/fvets.2026.1910014

**Published:** 2026-09-11

**Authors:** Abylay Sansyzbay, Uinkul Izbanova, Zaure Sayakova, Saltanat Nusupova, Dinara Kamalova, Akhmetzhan Sultanov, Mynbay Umitzhanov, Kairat Zhumanov, Serik Khizat, Alexandr Shevtsov, Nur Tukhanova

**Affiliations:** 1 Kazakh National Agrarian Research University, Almaty, Kazakhstan; 2 M. Aikimbayev's National Scientific Center for Especially Dangerous Infections, National Holding ≪QazBioPharm≫, Almaty, Kazakhstan; 3 Kazakh Scientific Research Veterinary Institute, National Holding ≪QazBioPharm≫, Almaty, Kazakhstan; 4 Laboratory of Applied Genetics, National Center for Biotechnology, National Holding ≪QazBioPharm≫, Astana, Kazakhstan; 5 Department Infectious and Tropical Disease, S.D. Asfendiyarov's Kazakh National Medical University, Almaty, Kazakhstan

**Keywords:** cox1 gene, *Hyalomma asiaticum*, *Hyalomma dromedarii*, *Hyalomma rufipes*, ixodid ticks, Kazakhstan, phylogenetic analysis, regional distribution records

## Abstract

**Background:**

Ticks of the genus *Hyalomma* are among the most important vectors of pathogens affecting humans and animals in arid and semi-arid regions of Eurasia. Recent environmental changes, including climate warming, increasing aridity, and livestock movements, may influence the distribution of *Hyalomma* species into previously unrecorded areas. However, information on the current distribution of these ticks in Kazakhstan remains incomplete.

**Methods:**

Field surveys were conducted in the Turkestan, Abai, and East Kazakhstan regions of Kazakhstan in 2024. A total of 137 tick specimens were collected from domestic animals and grouped into 47 pools. Tick species were identified using morphological characteristics and mitochondrial cytochrome c oxidase subunit I (*cox1*) gene sequencing. Species identification was confirmed by BLAST analysis against GenBank reference sequences and phylogenetic reconstruction using the Maximum-Likelihood method.

**Results:**

Morphological examination revealed the presence of *Hyalomma asiaticum, H. turanicum, H. dromedarii, H. rufipes*, and *Dermacentor marginatus*. Molecular analysis confirmed the identification of representative specimens of *H. dromedarii, H. rufipes*, and *H. asiaticum*, showing 100%, 99.8%, and 99.7% nucleotide identity, respectively, with corresponding GenBank sequences. Phylogenetic analysis grouped all samples within their respective species clades. Based on currently available published data, the study provides the first molecularly confirmed regional records of *H. dromedarii* and *H. rufipes* in the Turkestan region and the first molecularly confirmed regional record of *H. asiaticum* in East Kazakhstan region.

**Conclusions:**

These findings provide new documented distribution records for several *Hyalomma* species in Kazakhstan and improve current knowledge of their geographic occurrence. Continued surveillance will be necessary to determine whether these records represent recent range expansion or previously undocumented populations.

## 1 Introduction

Ticks of the genus *Hyalomma* Koch, 1844 (Acari: Ixodidae) are among the most important arthropod vectors inhabiting arid and semi-arid ecosystems, where they play a major role in the maintenance and transmission of pathogens affecting both humans and animals (1, 2). According to current taxonomic classifications, the genus *Hyalomma* comprises approximately 27 valid species distributed throughout Africa, the Middle East, Central Asia, and southern Europe (3). Several *Hyalomma* species are recognized vectors of a broad range of viral, bacterial, and protozoan pathogens of considerable medical and veterinary importance (1, 2).

In Kazakhstan, nine species of the genus *Hyalomma* have been reported, including *H. aegyptium, H. anatolicum, H. asiaticum, H. dromedarii, H. excavatum, H. marginatum, H. scupense, H. turanicum*, and *H. rufipes* (4, 5). Among them, *Hyalomma dromedarii* Koch, 1844 is strongly associated with camels and is considered a characteristic species of desert and semi-desert habitats throughout North Africa, the Middle East, and Central Asia (1, 6). *Hyalomma asiaticum* is one of the dominant tick species of Central Asian desert ecosystems and is involved in the circulation of several pathogens affecting livestock, wildlife, and humans (2, 4). *Hyalomma rufipes* is widely distributed in Africa and parts of Eurasia and has attracted increasing attention because of its capacity for long-distance dispersal via migratory birds (7–9).

The epidemiological significance of *Hyalomma* ticks has increased considerably during recent decades. Species of this genus are implicated in the transmission of numerous pathogens, including Crimean–Congo hemorrhagic fever virus (CCHFV), *Coxiella burnetii, Francisella tularensis, Rickettsia* spp., *Babesia* spp., *Theileria* spp., and other agents of zoonotic and veterinary importance (1, 2). In particular, *H. dromedarii* has been reported to harbor *Colpodella* spp., *Babesia* spp., members of the family Anaplasmataceae, and several species of spotted fever group rickettsiae (10–12).

In recent decades, climate change has been recognized as one of the major drivers of tick range expansion worldwide. Rising temperatures, shorter winter periods, and increasing aridity have facilitated the establishment of *Hyalomma* species in regions previously considered unsuitable for their survival (7, 13). In addition, movements of livestock, particularly camels, cattle, sheep, and horses, contribute to the dispersal of ticks over considerable distances. Migratory birds and wild mammals may also transport immature stages of *Hyalomma* ticks into new geographic areas, thereby facilitating the expantion of their geographic range (1, 2, 8).

Tick surveillance in livestock populations remains essential for understanding the distribution of medically and veterinary significant tick species and their associated pathogens (14). However, information on the current distribution of *Hyalomma* species in Kazakhstan remains incomplete. Recent surveys suggest that the ranges of several species may be changing, particularly in southern and eastern regions of the country (5). Detection of previously unrecorded populations is important for understanding the ecology of tick-borne pathogens and for improving surveillance programs targeting vector-borne diseases.

The aim of the present study was to determine the species composition of ixodid ticks collected from domestic animals in the Turkestan, Abai, and East Kazakhstan regions of Kazakhstan using morphological identification. Mitochondrial *cox1* sequencing was performed only for representative specimens corresponding to potentially new regional records in order to molecularly confirm their taxonomic identity.

## 2 Materials and methods

### 2.1 Study area and tick collection

Field sampling was conducted in the Turkestan, Abai, and East Kazakhstan regions of Kazakhstan between May and October 2024. Tick specimens were collected directly from domestic animals during epizootiological surveys.

Animals were carefully inspected for the presence of attached ticks, with particular attention paid to preferred attachment sites, including the ears, neck, axillary and inguinal regions, perineum, udder, and tail base. Ticks were removed manually using forceps and individually preserved in 70% ethanol for subsequent laboratory analyses. Verbal permission was obtained from the animal owners/farmers prior to examination and tick collection. For each collection, information on the host species, collection locality, date of collection, and attachment site was recorded.

Collected specimens were transported to the laboratory for morphological identification and subsequent laboratory analyses. Following morphological identification, tick specimens were grouped into pools according to species, individual host, and collection locality. Each pool contained 3–4 ticks in the Turkestan and Abai Regions and 1–2 ticks in the East Kazakhstan Region. Ticks belonging to different species, collected from different individual hosts, or originating from different localities were not combined within the same pool. These pools were prepared for pathogen screening as part of a separate investigation, pathogen-screening data are not included in the present study. The molecular taxonomic analyses reported here were performed on individually processed representative specimens, as described below.

### 2.2 Morphological identification

Morphological identification was performed using a stereomicroscope MBS 100T BIOLAB (China) according to standard taxonomic keys for ixodid ticks (6, 15, 16).

Species identification was based on diagnostic characters including the shape of the scutum, genital aperture, cervical grooves, spiracular plates, adanal and subanal shields, and caudal grooves.

Representative specimens were photographed using a digital microscope imaging system, and diagnostic morphological structures were documented.

### 2.3 Selection of specimens for molecular analysis

Following morphological identification, three individual specimens were selected for molecular confirmation: one specimen morphologically identified as *Hyalomma dromedarii* and one as *H. rufipes* from the Otyrar district, Turkestan region, and one specimen morphologically identified as *H. asiaticum* from the Zaisan district, East Kazakhstan region. Of the three *H. asiaticum* specimens collected in the Zaisan district (two females and one male), one representative specimen was selected for molecular analysis.

The three selected specimens were processed individually rather than as pooled samples. Each specimen was subjected separately to DNA extraction, PCR amplification, and *cox1* sequencing. Molecular confirmation was not performed for specimens morphologically identified as *H. turanicum* or *Dermacentor marginatus*, as these species are known components of the tick fauna in the respective study regions and did not represent focal regional distribution records in the present study. Molecular analysis was therefore targeted at representative specimens of *H. dromedarii, H. rufipes*, and *H. asiaticum* associated with the regional distribution records investigated here.

### 2.4 DNA extraction and PCR amplification

Genomic DNA was extracted using the D-Cells DNA extraction kit (Biolabmix, Russia) according to the manufacturer's instructions. A fragment of the mitochondrial cytochrome c oxidase subunit I (*cox1*) gene was amplified using the universal primers Cox1-F (5′-GGAACAATATATTTAATTTTTGG-3′) and Cox1-R (5′-ATCTATCCCTACTGTAAATATATG-3′) (17).

PCR amplification was performed in a final reaction volume of 25 μL containing 12.5 μL of 2 × BioMaster HS-Taq PCR-Color Mix (Biolabmix, Russia), 1 μL (10 pmol) of each primer, 5 μL of DNA template, and 5.5 μL of nuclease-free water. Amplification was carried out using a Eppendorf Mastercycler^®^ X50 (Eppendorf, Germany) under the following conditions: initial denaturation at 95 °C for 5 min; 45 cycles of denaturation at 95 C for 30 s, annealing at 63°C for 30 s, and extension at 72°C for 50 s; followed by a final extension at 72°C for 5 min.

### 2.5 DNA sequencing and analysis

The amplified PCR products were separated by electrophoresis in a 1.5% agarose gel stained with ethidium bromide. The resulting PCR amplicons were purified using magnetic particles as previously described (18). Sanger sequencing was performed using the Big Dye Terminator v3.1 Cycle Sequencing Kit (Thermo Fisher Scientific, Vilnius, Lithuania) according to the manufacturer's instructions. Sequencing reaction products were resolved on a GeneticAnalyzer 3730 xl (Applied Biosystems, Carlsbad, CA, USA). Sequences from the two strands were assembled using SeqMan (Lasergene, DNASTAR) (19).

The obtained nucleotide sequences were compared with those available in the GenBank database using the BLASTn algorithm to confirm species identity. To improve the reliability of molecular identification, multiple representative *cox1* reference sequences of each target species were retrieved from GenBank from different geographic regions and published studies. Sequences of closely related *Hyalomma* species were also included in the phylogenetic analysis.

**Figure 1:**
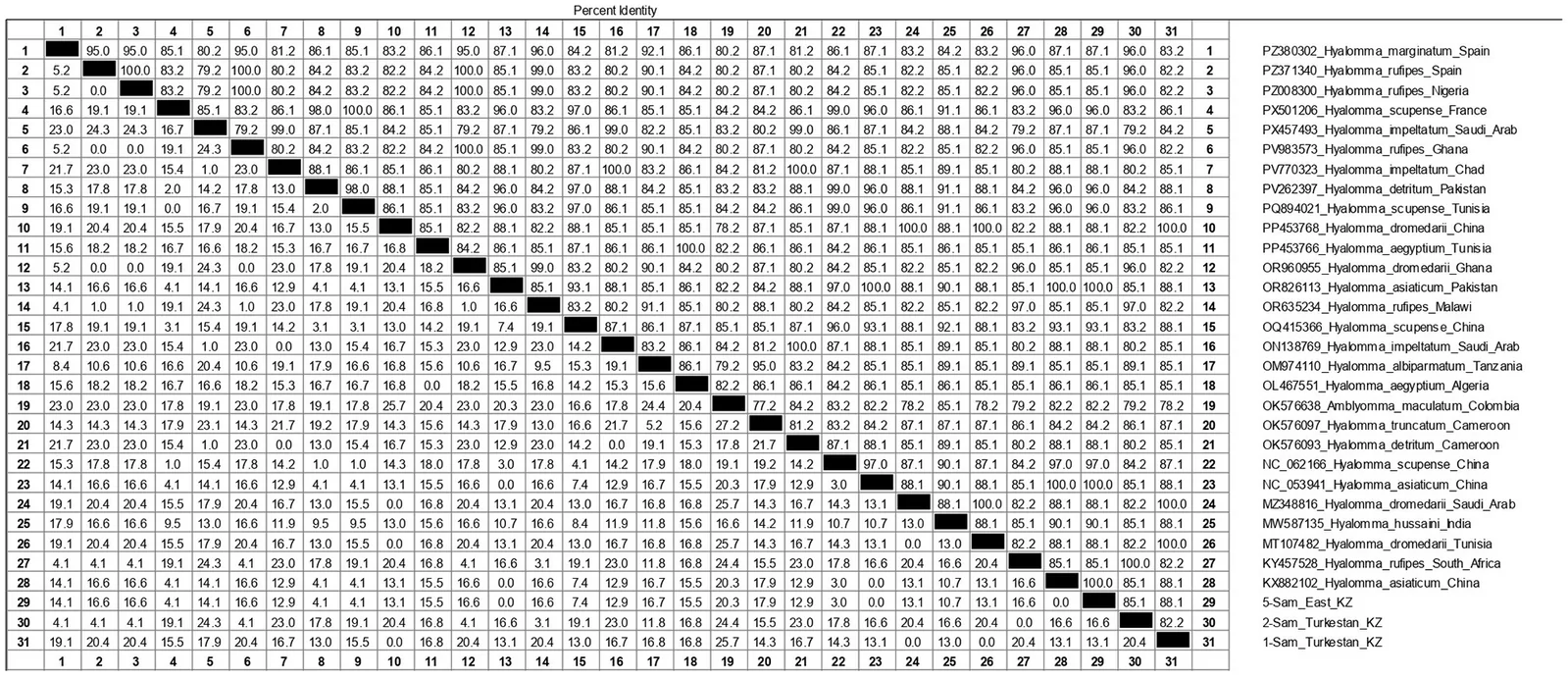
Pairwise nucleotide identity matrix based on the multiple alignment of partial mitochondrial *cox1* sequences from the Kazakhstan *Hyalomma* specimens and selected GenBank references. Values were calculated from homologous positions retained in the alignment and should therefore be interpreted separately from the BLASTn identities reported in Table 1.

Multiple sequence alignment was performed using ClustalW implemented on MEGA 11 (version 11.0.13). A Maximum Likelihood analysis was performed in MEGA 11 under Kimura 2-parameter model with 1,000 bootstrap replicates. Bootstrap values were used to assess the reliability of the inferred tree topology. The phylogenetic tree was visualized using MEGA 11 (20). Multiple sequence alignment was performed using ClustalW implemented in MEGA 11 (version 11.0.13). Pairwise nucleotide identities presented in Figure 1 were calculated from the aligned sequences in MEGA 11. BLASTn identity values and alignment-based pairwise identity values were interpreted separately because they were obtained using different analytical procedures and may involve different comparison lengths and reference sequences. Phylogenetic reconstruction was performed using the Maximum Likelihood method in MEGA 11 under the Kimura 2-parameter substitution model with 1,000 bootstrap replicates. Bootstrap values were used to assess the statistical support of the inferred tree topology. The phylogenetic tree was visualized in MEGA 11 (20).

## 3 Results

### 3.1 Tick collection and species composition

**Figure 2:**
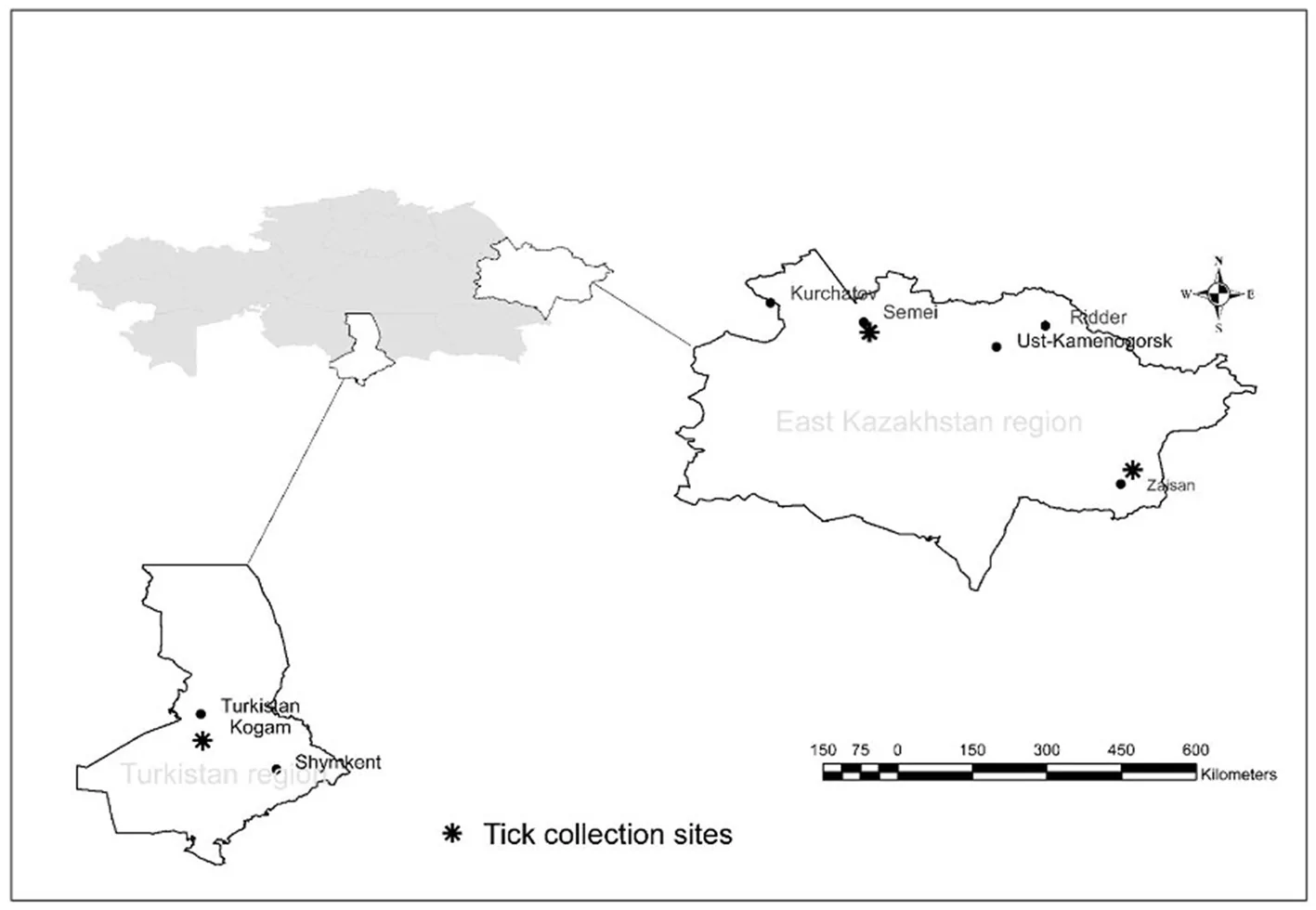
Geographic locations of tick collection sites in the Turkestan, Abai, and East Kazakhstan regions of Kazakhstan. Sampling sites included the Otyrar district (Kogam village) in the Turkestan region, Zhanasemei district in the Abai region, and the Zaisan district in the East Kazakhstan region.

During the study period, a total 50 domestic animals were examined for tick infestation across the three study regions. In the Turkestan region, 15 camels were examined, in the Abai region 20 cattle were examined, and in the East Kazakhstan region 15 cattle were examined. A total of 137 tick specimens were collected, including 49 ticks from camels in the Turkestan region, 68 ticks from cattle in the Abai region, and 20 ticks from cattle in the East Kazakhstan region (Figure 2).

Morphological examination identified five ixodid tick species belonging to two genera. In the Turkestan Region, *Hyalomma asiaticum, H. turanicum, H. dromedarii*, and *H. rufipes* were identified, whereas *Dermacentor marginatus* was identified in the Abai Region. In the East Kazakhstan Region, *D. marginatus* and *H. asiaticum* were recorded.

The identifications of *H. turanicum* and *D. marginatus* were based exclusively on morphological examination. Molecular confirmation was performed on representative specimens of *H. dromedarii, H. rufipes*, and *H. asiaticum*.

### 3.2 Morphological identification of *Hyalomma dromedarii*

Among the collected specimens, several adult ticks removed from camels in the Kogam rural district of the Otyrar district were morphologically identified as *H. dromedarii* Koch, 1844. Morphologically, *H. dromedarii* closely resembles *H. asiaticum* and *H. scupense*, both of which occur in the study region and may also parasitize camels. However, the collected specimens could be distinguished from these species by several diagnostic characters.

**Figure 3:**
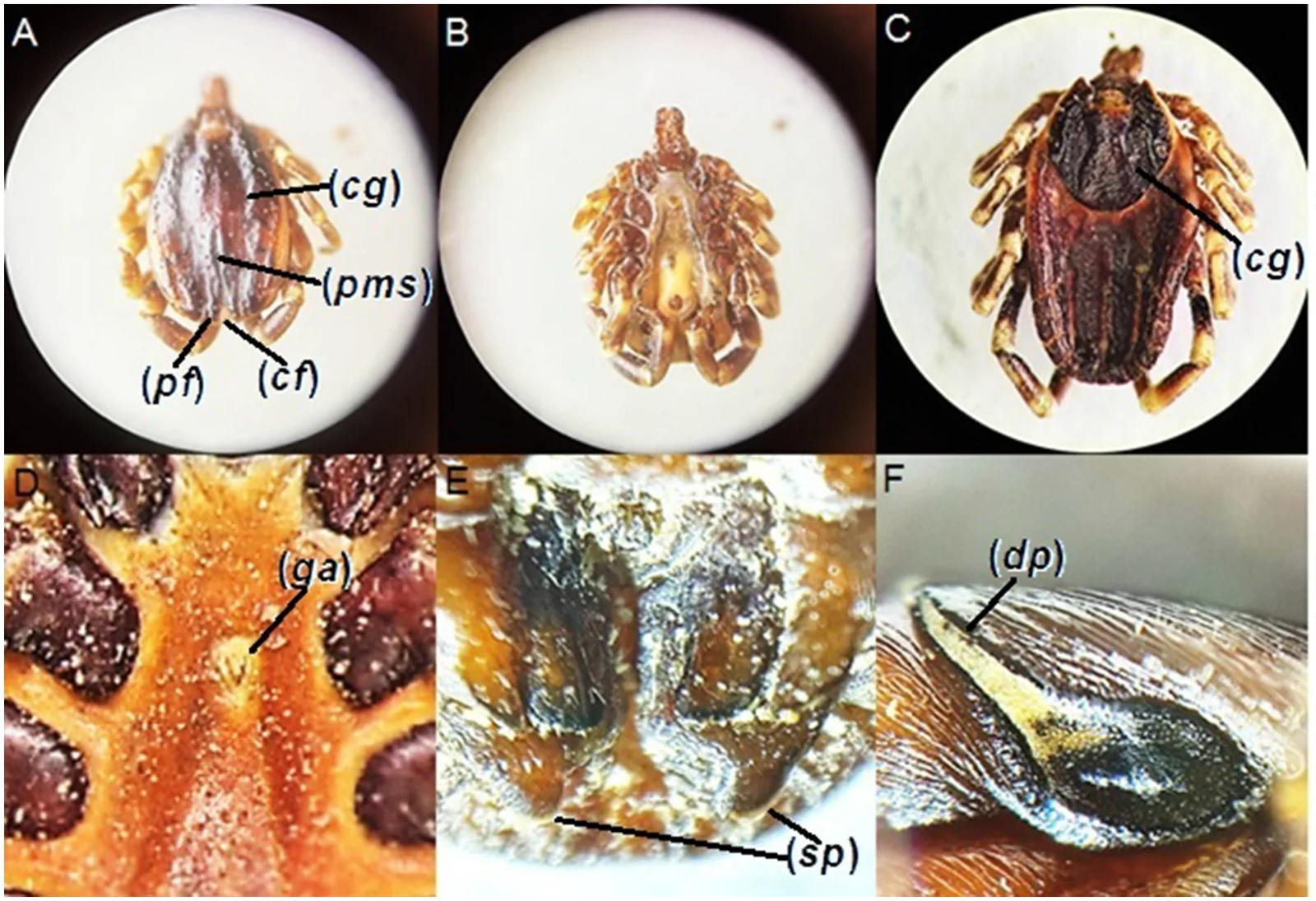
Morphological characteristics of *Hyalomma dromedarii* collected from camel in the Otyrar district, Turkestan region, Kazakhstan: **(A)** male, dorsal view: cervical groove (cg), posteromedian groove (pms), central festoon (parma) (cf), paracentral festoon (pf); **(B)** male, ventral view; **(C)** female, dorsal view: cervical groove (cg); **(D)** female, ventral view: genital aperture (ga); **(E)** male, ventral view: subanal plate (sp); **(F)** male, spiracular plate: dorsal process (dp).

Male *H. dromedarii* differed from *H. asiaticum* by the presence of a long and deep median caudal groove extending to the parma (Figures 3A, B), whereas the groove does not reach the parma in *H. asiaticum*. The males also possessed large plate-like subanal shields located posterior to the accessory shields (Figure 3E), and relatively short dorsal prolongation of the spiracular plate (Figure 3F). Females were characterized by a broadly rounded posterior margin of the scutum (Figure 3C) and a flat V-shaped genital aperture (Figure 3D), whereas in *H. asiaticum* the genital aperture is convex and U-shaped.

The examined specimens also differed from *H. scupense*. In male *H. dromedarii*, the cervical grooves were long and deep (Figure 3A, B), similar to those of *H. asiaticum*, the subanal shields were broad, and the dorsal prolongation of the spiracular plate was relatively narrow (Figure 3F). In contrast, male *H. scupense* possess short and shallow cervical grooves, narrow apically pointed subanal shields located posterior to the adanal shields, and a broad dorsal prolongation of the spiracular plate. Females of *H. scupense* are characterized by a flat, rounded genital aperture.

### 3.3 Morphological identification of *Hyalomma rufipes*

A single male specimen of *H. rufipes* Koch, 1844 was collected from a camel in the Kogam rural district of the Otyrar district, Turkestan region, southern Kazakhstan.

**Figure 4:**
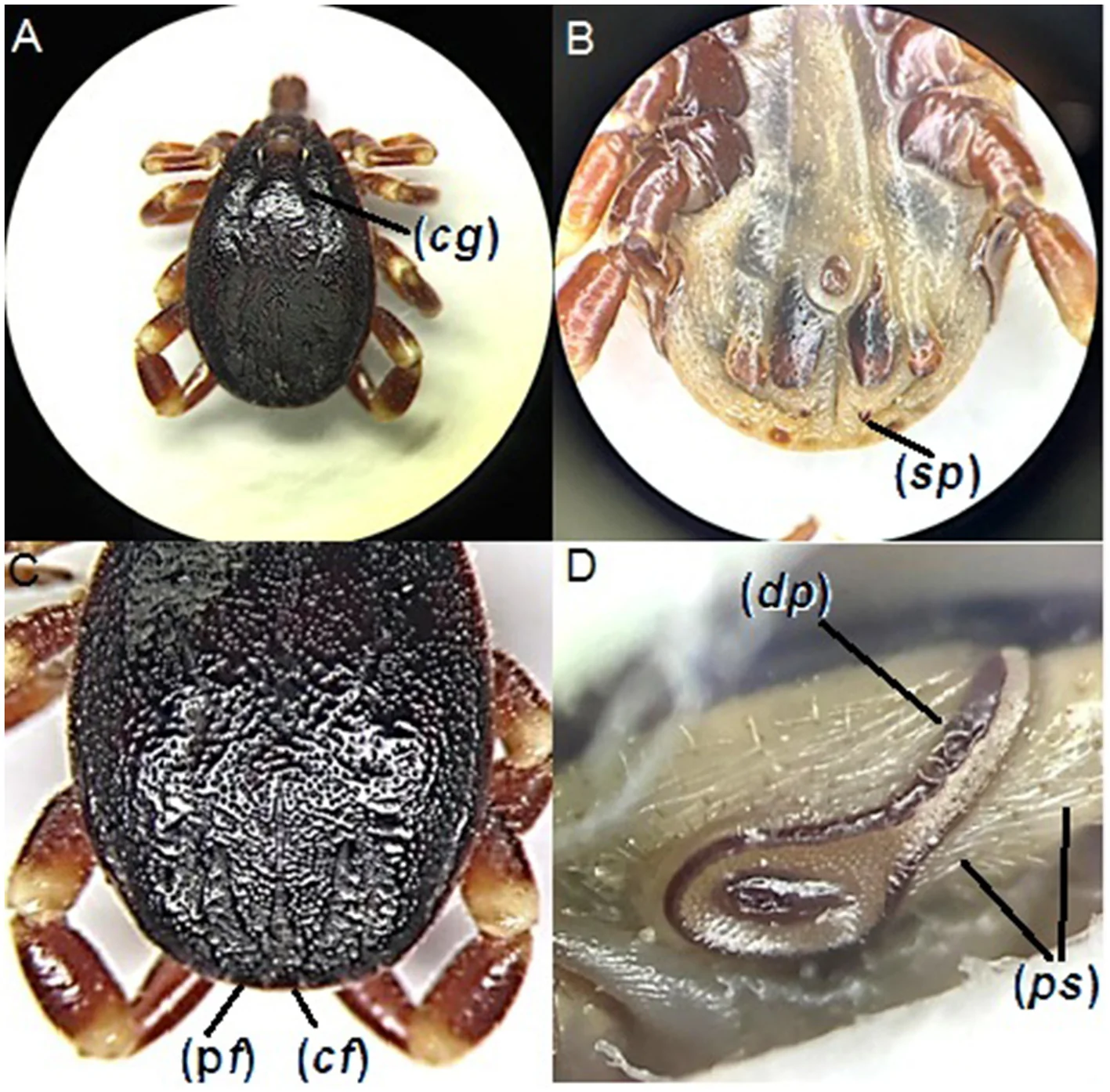
Morphological characteristics of *Hyalomma rufipes* collected from camel in the Otyrar district, Turkestan region, Kazakhstan: **(A)** male, dorsal view: cervical groove (cg); **(B)** Male ventral view: subanal plate (sp); **(C)** Male, posterior region: central festoon (cf), paracentral festoon (pf); **(D)** Male, peritreme: dorsal process (dp), peritremal setae (ps).

In terms of morphological features, *H. rufipes* is very similar to *H. marginatum*, which inhabits western Kazakhstan, and *H. turanicum*, which is found in the study region. These species share similar features: short cervical and posteromedian grooves (Figure 4A), short postero-median grooves (Figure 4C), and a central festoon (parma) that is not wider than the adjacent paracentral festoons (Figure 4B). However, the examined specimen differed from these species in several diagnostic characters. The male exhibited very dense and fine punctation uniformly distributed over the entire conscutum (Figure 4C). The dorsal prolongation of the peritreme was narrow (Figure 4D), being narrower than that of *H. turanicum* and much narrower than that of *H. marginatum*.

### 3.4 Morphological identification of *Hyalomma asiaticum*

Three adult specimens of *H. asiaticum* Schulze & Schlottke, 1930 (two females and one male) were collected from cattle in the Zaisan district of East Kazakhstan region. Diagnostic morphological characters of the examined specimens are shown in Figure 4.

**Figure 5:**
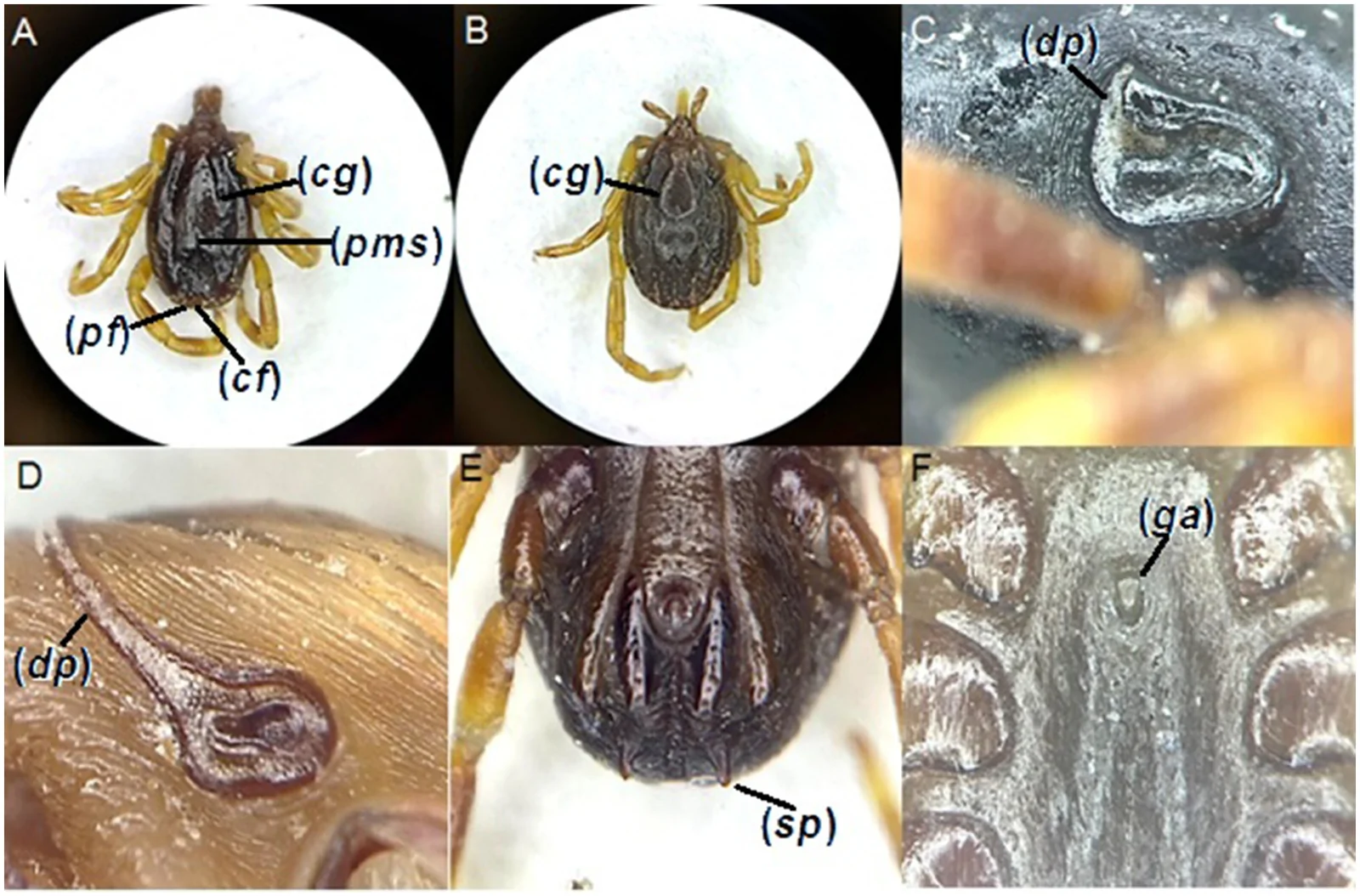
Morphological characteristics of *Hyalomma asiaticum* collected from cattle in the Zaisan district, East Kazakhstan region, Kazakhstan: **(A)** male, dorsal view: cervical groove (cg), posteromedian groove (pms), central festoon or parma (cf), paracentral festoon (pf); **(B)** female, dorsal view: cervical groove (cg); **(C)** female, spiracular plate: dorsal process (dp); **(D)** male, peritreme: dorsal process (dp); **(E)** male, ventral view: subanal plate (sp); **(F)** female, ventral view: genital aperture (ga).

Males were distinguished by long, deep cervical grooves (Figure 5A), well-developed adanal and subanal shields (Figure 5E), and a long, narrow dorsal prolongation of the peritreme (Figure 5D). The parma (central festoon) was light-colored and wider than the adjacent festoons (Figure 5E). The median caudal groove did not reach the parma (Figure 5E), which is a characteristic feature distinguishing *H. asiaticum* from *H. dromedarii*.

In females, the scutum exhibited deep cervical grooves extending toward the posterior margin and narrowing posteriorly (Figure 5B), whereas the genital aperture was convex and U-shaped (Figure 5F). The morphology of the spiracular plate (Figure 5C) and other diagnostic structures was consistent with published descriptions of *H. asiaticum* (15).

Based on the combination of these morphological characters, all examined specimens were identified as *H. asiaticum*. Subsequent molecular analysis of the mitochondrial *cox1* gene confirmed the morphological identification.

This finding represents the first documented record of *H. asiaticum* in East Kazakhstan region. Previously, the eastern distribution limit of the species in Kazakhstan was considered to be the Zhetysu (Dzungarian) Alatau mountain system. No confirmed records east of this boundary have been reported in the available literature.

The present record extends the known distribution range of *H. asiaticum* eastward and indicates a possible ongoing expansion of the species within Kazakhstan.

### 3.5 Molecular identification and phylogenetic analysis

PCR amplification of the mitochondrial *cox1* gene produced amplicons of approximately 820 bp for all three representative specimens selected for molecular analysis. No amplification was observed in the negative control, indicating the absence of detectable contamination.

Following quality trimming and removal of primer sequences, the final *cox1* consensus sequences were 453 bp for Sample 1 (*H. dromedarii*), 590 bp for Sample 2 (*H. rufipes*), and 704 bp for Sample 5 (*H. asiaticum*). The sequences were deposited in GenBank under accession numbers PZ241075 (*H. dromedarii*, Sample 1), PZ241076 (*H. rufipes*, Sample 2), and PZ241077 (*H. asiaticum*, Sample 5), respectively.

BLASTn analysis supported the morphological identification of all three specimens. Based on the final quality-trimmed partial mitochondrial *cox1* sequences, Sample 1 was identified as *H. dromedarii*, showing 100.0% nucleotide identity to its closest GenBank match. Sample 2 was identified as *H. rufipes* with 99.8% nucleotide identity, whereas Sample 5 was identified as *H. asiaticum* with 99.7% nucleotide identity to their respective closest GenBank matches (Table 1).

**Table 1:** Molecular identification of *Hyalomma* tick specimens based on mitochondrial *cox1* gene sequences and BLAST analysis against GenBank reference sequences.

Sample ID	Species	Region	Sequence length (bp)	BLAST Identity (%)	GenBank accession number
Sample 1	*H. dromedarii*	Otyrar district, Turkestan region	453	100	PZ241075
Sample 2	*H. rufipes*	Otyrar district, Turkestan region	590	99.8	PZ241076
Sample 5	*H. asiaticum*	Zaisan district, East Kazakhstan region	704	99.7	PZ241077

Pairwise sequence comparison based on the multiple-sequence alignment further demonstrated a high level of nucleotide similarity between the Kazakhstan specimens and selected conspecific GenBank reference sequences (Figure 1). Some individual sequence pairs showed 100% identity over the homologous positions retained in the alignment. These alignment-based pairwise values were interpreted separately from the BLASTn identities reported in Table 1 because the two analyses differed in the length of the compared regions and, in some cases, in the reference sequences used.

**Figure 6:**
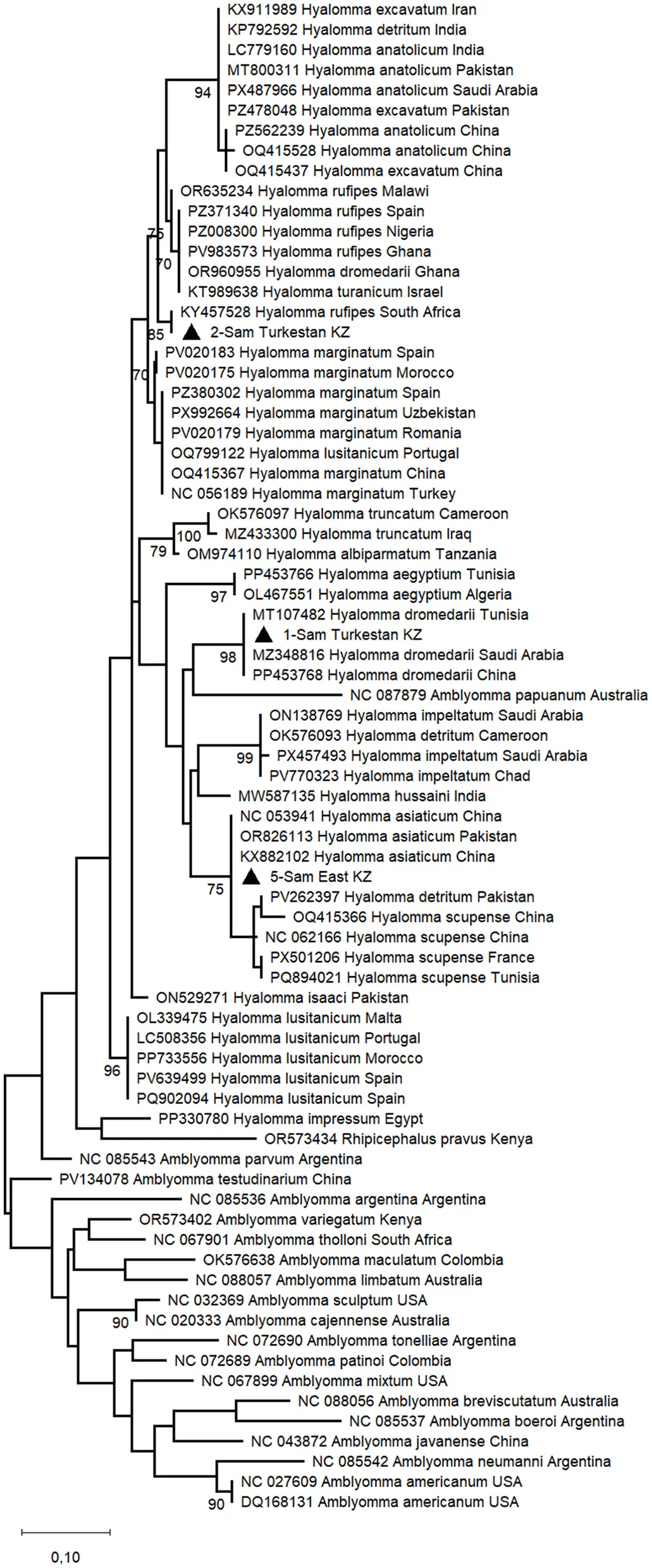
Maximum Likelihood phylogenetic tree based on partial mitochondrial *cox1* gene sequences of *Hyalomma* ticks collected in Kazakhstan and representative reference sequences retrieved from GenBank. Evolutionary distances were estimated using the Maximum Composite Likelihood model. Sequences generated in the present study are indicated by black triangles (▴). Sample 1, Sample 2, and Sample 5 correspond to the labels 1-Sam, 2-Sam, and 5-Sam, respectively, in the phylogenetic tree. Sample 1 clustered with *Hyalomma dromedarii*, Sample 2 with *H. rufipes*, and Sample 5 with *H. asiaticum*. Bootstrap support values (1,000 replicates) are shown at the nodes. Species of the genus Amblyomma were used as outgroups.

Maximum Likelihood phylogenetic analysis of partial mitochondrial *cox1* sequences further supported the taxonomic placement of the Kazakhstan *Hyalomma* specimens. Sample 1 clustered with reference sequences of *H. dromedarii* from China (PP453768), Saudi Arabia (MZ348816), and Tunisia (MT107482). Sample 2 clustered most closely with the South African *H. rufipes* reference sequence KY457528, consistent with its morphological identification and BLASTn assignment. Sample 5 grouped with representative *H. asiaticum* sequences from China (KX882102) and Pakistan (OR826113). Overall, the BLASTn, pairwise sequence comparison, and phylogenetic results were concordant with the morphological identification of the examined specimens (Figure 6).

### 3.6 Distributional records of *Hyalomma* species

The present study documents three noteworthy distributional records of *Hyalomma* ticks in Kazakhstan. *H. dromedarii* and *H. rufipes* were recorded in the Otyrar district of the Turkestan region, whereas *H. asiaticum* was detected in the Zaisan district of East Kazakhstan region.

To our knowledge, this represents the first molecularly confirmed record of *H. dromedarii* in the Turkestan region, the first documented record of *H. rufipes* in the Turkestan region, and the first documented record of *H. asiaticum* in East Kazakhstan region. These records expand the currently documented distribution of the respective species within Kazakhstan and provide new data on the geographic occurrence of *Hyalomma* ticks in the country. Based on the currently available published literature, these findings represent the first documented regional records; however, additional long-term surveillance is required to determine whether they reflect recent range expansion or previously undocumented populations.

## 4 Discussion

The present study provides molecular and morphological evidence of previously undocumented occurrences of three epidemiologically important *Hyalomma* species in Kazakhstan. Based on the currently available published literature, these findings represent the first documented regional records of *H. dromedarii, H. rufipes* in the Turkestan region, and *H. asiaticum* in the East Kazakhstan region. These findings improve our understanding of the current regional distribution of *Hyalomma* ticks.

Among the findings reported here, the detection of *H. dromedarii* in the Turkestan region is of particular importance. This species is primarily associated with camels and is considered one of the characteristic tick species of desert and semi-desert ecosystems (1, 6). Previous records in Kazakhstan were largely restricted to western regions, particularly Mangystau (5). Based on the currently available published records, the present study represents the first molecularly confirmed record of *H. dromedarii* in the Turkestan region and expands the documented distribution of this species within Kazakhstan. The occurrence of *H. dromedarii* in southern Kazakhstan may be associated with livestock movements, particularly those involving camels, increasing suitability of local climatic conditions, or previously undetected populations. Because this species is a recognized vector of CCHFV, *Coxiella burnetii, Francisella tularensis, Rickettsia* spp., and several protozoan pathogens (2, 12, 21, 22), continued monitoring of camel-associated tick populations is warranted.

The first documented regional record of *H. rufipes* in the Turkestan region is also noteworthy. *Hyalomma rufipes* is a two-host tick species commonly associated with arid and semi-arid environments and is widely distributed throughout sub-Saharan Africa, parts of the Middle East, and Eurasia (15, 23). The species is well known for its ability to disperse over long distances via migratory birds carrying immature stages (7, 9). Consequently, occasional records outside its traditional range have been reported from several European and Asian countries (8, 24–26). The occurrence of *H. rufipes* in southern Kazakhstan may reflect accidental introduction by migratory birds, recent establishment of local populations, or previous under-detection. Additional surveys will be necessary to determine whether stable populations of the species have become established in the region. The detection of *H. rufipes* in Kazakhstan is of considerable epizootological and epidemiological significance, as migratory birds may transport immature stages of this tick together with associated microorganisms over long distances, potentially contributing to the movement of rickettsiae and other tick-associated bacteria between Africa and the Palearctic region.

The detection of *H. asiaticum* in East Kazakhstan region is particularly noteworthy because this species has traditionally been associated with desert and semi-desert ecosystems of western and southern Kazakhstan (4). The eastern limit of its known distribution was previously considered to correspond to the Zhetysu (Dzungarian) Alatau mountain system. Based on the currently available published literature, the present finding represents the first documented regional record of *H. asiaticum* in the East Kazakhstan region and extends the currently documented distribution of the species within Kazakhstan. Considering the proximity of the Zaisan district to the border with China, dispersal involving small mammals, particularly rodents, cannot be excluded. Rodents are recognized hosts of immature *H. asiaticum* and may contribute to transboundary dispersal of the species.

Several factors may contribute to the occurrence of these species in regions where they had not previously been documented. Climatic warming, increasing aridity, changes in land use, livestock movements, and dispersal by migratory birds and wild mammals have been identified as important drivers of changes in the geographic distribution of *Hyalomma* species throughout Eurasia (7, 13). Similar processes may contribute to the occurrence of *H. dromedarii, H. rufipes*, and *H. asiaticum* in the regions investigated here. The present findings extend the documented geographic distribution of *H. dromedarii* and *H. rufipes* to the Turkestan region and of *H. asiaticum* to the East Kazakhstan region, based on currently available published records. However, these observations should not be interpreted as direct evidence of ongoing geographic range expansion. Because sampling was conducted during a single collection period, it is not possible to determine whether these records reflect recent geographic expansion or introduction, establishment of local populations, or previously undetected occurrence. Long-term surveillance incorporating repeated sampling across multiple seasons and years will be required to determine temporal changes in the distribution of these species.

The detection of previously unrecorded *Hyalomma* species highlights the importance of continuous entomological surveillance in Kazakhstan. Changes in the distribution of vector species may alter the epidemiology of tick-borne infections and facilitate the emergence of pathogens in previously unaffected areas. Integration of classical morphological identification with molecular approaches provides a reliable framework for monitoring tick distributions and improving risk assessment for vector-borne diseases in Central Asia.

Alignment-based pairwise sequence comparison provided additional support for the taxonomic placement of the Kazakhstan specimens. Some comparisons yielded 100% nucleotide identity over the homologous cox1 positions included in the alignment. Specifically, Sample 2 showed 100% identity to the South African *H. rufipes* sequence KY457528, Sample 1 to the *H. dromedarii* sequences from Saudi Arabia (MZ348816) and Tunisia (MT107482), and Sample 5 to the *H. asiaticum* sequences from China (NC_053941 and KX882102). These alignment-based pairwise values should be distinguished from the BLASTn identities of 100.0%, 99.8%, and 99.7%, which were calculated independently using the complete quality-trimmed query sequences and are reported in Table 1. The slight differences between the two analyses reflect differences in the compared sequence regions and, in some cases, in the reference sequences used, and do not indicate conflicting taxonomic assignments.

However, the broader phylogenetic analysis revealed complex relationships among some sequences assigned to different nominal *Hyalomma* species. In particular, *H. rufipes* reference sequences were recovered in separate lineages, and some sequences assigned to different nominal species occurred within closely related clades. These patterns may reflect the limited resolution of the partial mitochondrial *cox1* marker, misidentification or inconsistent taxonomic annotation of publicly available sequences, unresolved species boundaries, cryptic diversity, incomplete lineage sorting, or mitochondrial introgression. The present dataset does not allow these alternative explanations to be distinguished; therefore, the observed topology cannot be attributed to a single mechanism. Further studies incorporating broader taxon sampling, additional mitochondrial and nuclear markers, and analyses of intra- and interspecific genetic distances are needed to clarify species boundaries and phylogenetic relationships within *Hyalomma*.

This study has several limitations. First, molecular characterization was performed on representative specimens corresponding to the focal regional records rather than on all collected specimens. Specifically, molecular confirmation was restricted to representative specimens of *H. dromedarii, H. rufipes*, and *H. asiaticum*, whereas *H. turanicum* and *Dermacentor marginatus* were identified exclusively on the basis of morphological characters. Therefore, the molecular dataset should not be interpreted as molecular validation of the complete tick species assemblage observed during the survey. The molecular component of the study was designed primarily to provide independent confirmation of the taxonomic identity of specimens associated with the focal regional records rather than to characterize all species collected or to evaluate intraspecific genetic diversity and population genetic structure. Second, sampling was restricted to three regions of Kazakhstan during a single collection period, which may not fully capture the spatial and temporal distribution of *Hyalomma* species across the country. Finally, molecular identification relied exclusively on partial mitochondrial *cox1* sequences of unequal length. In particular, the 453-bp sequence obtained for *H. dromedarii* was shorter than the other sequences analyzed and may provide reduced phylogenetic resolution. Therefore, the phylogenetic topology should be interpreted cautiously, particularly with respect to relationships among closely related *Hyalomma* taxa. Species-level identification in the present study was based on concordance between morphological characters, BLASTn comparisons, pairwise sequence comparisons, and phylogenetic placement rather than on tree topology alone. Future studies incorporating larger numbers of specimens per species, broader geographic and seasonal sampling, additional mitochondrial and nuclear markers, analyses of intra- and interspecific genetic distances, and more comprehensive phylogenetic approaches will provide a more complete understanding of species boundaries, genetic diversity, population structure, and phylogeographic relationships among *Hyalomma* ticks in Kazakhstan.

## 5 Conclusion

This study documents new regional records of *H. dromedarii* and *H. rufipes* in the Turkestan region and *H. asiaticum* in the East Kazakhstan region based on currently available published data. Morphological identification supported by targeted *cox1* sequencing provides evidence for the occurrence of these species in the respective regions and extends their documented distribution within Kazakhstan. However, the present cross-sectional data do not establish whether these records represent recent range expansion or previously undetected populations. Continued systematic and longitudinal surveillance is therefore needed to determine whether these species are locally established and to assess temporal changes in their geographic distribution.

## Data Availability

The partial mitochondrial *cox1* sequences generated in this study have been deposited in GenBank under accession numbers PZ241075 (*Hyalomma dromedarii*, Sample 1), PZ241076 (*H. rufipes*, Sample 2), and PZ241077 (*H. asiaticum*, Sample 5).
